# Historical Changes in Honey Bee Wing Venation in Romania

**DOI:** 10.3390/insects12060542

**Published:** 2021-06-10

**Authors:** Adam Tofilski, Eliza Căuia, Adrian Siceanu, Gabriela Oana Vișan, Dumitru Căuia

**Affiliations:** 1 Department of Zoology and Animal Welfare, University of Agriculture in Krakow, Al. 29 Listopada 54, 31-425 Krakow, Poland; 2 Honeybee Genetics and Breeding Laboratory, Institute for Beekeeping Research and Development, Blv Ficusului, No. 42, Sector 1, 013975 Bucharest, Romania; eliza.cauia@icdapicultura.ro (E.C.); siceanu.adrian@gmail.com (A.S.); gabrielaoana81@gmail.com (G.O.V.); dumitru.cauia@icdapicultura.ro (D.C.)

**Keywords:** honey bee, *Apis mellifera*, wing, venation, Romania

## Abstract

**Simple Summary:**

Honey bees, in addition to producing honey, are important pollinators of wild and cultivated plants. Unfortunately, in some places, the population of honey bees is declining. One of the factors that affect their survival is adaptation to the local environment. Bees native to a particular area survive better than those imported. Despite this fact, some beekeepers import non-native bees and use them in their apiaries. Imported bees produce hybrids with bees from surrounding colonies because beekeepers do not control their mating. In consequence, the whole population can change. In this study, we verified how the population of Romanian bees has changed over the last four decades. We found significant temporal changes in wing venation. Despite these changes, the two major subpopulations of bees separated by mountains remain distinct. We provide a tool for the easy identification of native bees from Romania, which can help to protect them.

**Abstract:**

The honey bee (*Apis mellifera*) is an ecologically and economically important species that provides pollination services to natural and agricultural systems. The biodiversity of the honey bee is being endangered by the mass import of non-native queens. In many locations, it is not clear how the local populations have been affected by hybridisation between native and non-native bees. There is especially little information about temporal changes in hybridisation. In Romania, *A. m. carpatica* naturally occurs, and earlier studies show that there are two subpopulations separated by the Carpathian Mountains. In this study, we investigated how the arrangement of veins in bees’ wings (venation) has changed in Romanian honey bees in the last four decades. We found that in the contemporary population of Romanian bees, there are still clear differences between the intra- and extra-Carpathian subpopulations, which indicates that natural variation among honey bees is still being preserved. We also found significant differences between bees collected before and after 2000. The observed temporal changes in wing venation are most likely caused by hybridisation between native bees and non-native bees sporadically introduced by beekeepers. In order to facilitate conservation and the monitoring of native Romanian bees, we developed a method facilitating their identification.

## 1. Introduction

Honey bees (*Apis mellifera*) are important pollinators of wild and cultivated plants [1]. Their native distribution range extends from Scandinavia in the north to the Cape of Good Hope in the south, and from Portugal in the west to China in the east. Within this wide span, there are more than 20 subspecies (geographical races) [2,3,4,5,6]. The subspecies are grouped into 4–6 evolutionary lineages [6,7,8]. It is generally accepted that some subspecies are being endangered by hybridisation [9,10,11]. This hybridisation is mainly related to the importation of non-native honey bee subspecies (in particular *A. m. carnica*, *A. m. ligustica* and their hybrids) and the intensive proliferation of their genes by queen breeding [4,9]. The introgression caused by the introduction of non-native honey bees is a serious problem because their natural mating is not controlled by beekeepers [12,13,14]. Mating occurs in drone congregation areas, where queens and drones come from relatively large areas. Honey bee queens can perform several mating flights, during which they mate in the air with up to 28 (average 17) drones [15]. In consequence, a single non-native colony can produce hybrids with surrounding colonies, both managed and feral, within relatively large distances of up to 15 km [13,16,17]. This problem is particularly acute in Europe, where beekeeping is intensive [18], and feral populations are relatively small [19,20,21].

In large parts of Europe, there is a problem with hybridisation between native and non-native bees [21,22,23]. Therefore, the scientific community constantly advocates for the preservation of the diverse genetic resources of locally adapted bees, as locally adapted subspecies and ecotypes are more likely to survive in the presence of various stressors [24]. This is especially important for the breeding and selection activities in the light of the “conservation by utilization” principle [25].

Monitoring hybridisation between native and non-native subspecies is vital to the protection of local subspecies and ecotypes [26]. It is important to know not only if hybridisation is present but also how fast it is progressing. Unfortunately, there is very little information about the dynamics of hybridisation. Even if the same geographical locations were inspected repeatedly over time, methodologies frequently differ markedly between studies. Older studies were often based on morphology [6], while recent studies are more commonly based on molecular markers [5]. Even if the approaches detect some hybridisation, it is difficult to conclude whether or not introgression is progressing.

Most of the studies of temporal changes in honey bees were conducted outside their native range. These studies mainly documented the replacement of European bees with invasive Africanised bees [27,28,29] on American continents or reported differences between bees used by honey bee queen breeders in the USA [30]. Some studies based on allozyme frequencies did not detect significant differences over periods of a few years [31]. Studies based on whole-genome sequencing [32,33] detected clear temporal differences. In the Azores, where honey bees were probably introduced by colonisers [34], changes in mitotype composition varied between individual islands [35]. In some of these, there were marked increases in bee populations from Lineage C [35].

There is little information about temporal changes in native honey bee populations in mainland Europe. One of the studies based on the whole-genome sequencing of museum and contemporary specimens investigated the genetic diversity of *A. m. mellifera* in Switzerland [36]. From this survey alone, it is not possible to estimate the level of admixture of non-native subspecies because contemporary samples consisted of selected individuals that were the most similar to native subspecies. However, in combination with another study [37], and assuming random sampling, it can be estimated that in Switzerland, the admixture increased from 4.5–9.1% before 1959 to 31–60% in 2016. It was also observed that, over time, honey bees in Malta become less similar to Lineage A and more similar to Lineage C [38].

Studies of temporal changes are particularly interesting when involving borders between two subpopulations or ecotypes. Such a situation pertains to Romania, where the Carpathian Mountains form a natural barrier between the intra-Carpathian population in the northwest and the extra-Carpathian population in the rest of the country. Differences between the various populations of Romania were investigated in the first half of the 20th century [39]. Honey bees from Romania are distinct from bees in neighbouring countries, so they were designated as a separate subspecies—*Apis mellifera carpatica* [40]. However, Ruttner [6] suggested that in the intra-Carpathian area of Romania would be *A. m. carnica* and in the extra-Carpathian area *A. m. macedonica*. Earlier studies of *A. m. carpatica* were based on wing size and the cubital index (which is a ratio of two wing vein lengths of the cubital cell) [39,40]. Later, it was investigated on the basis of mitochondrial DNA [41,42,43], microsatelites [44] and single-nucleotide polymorphism [45]. *A. m. carpatica* belongs to Lineage C [2,42,46], and it can be identified using molecular methods [45,47]. It differs from other subspecies not only in morphology but also in physiology [48] and behaviour [40]. In this study, we used geometric morphometrics of honey bee wings because it is a reliable method of subspecies identification, it does not require sophisticated equipment and it could be applied by beekeepers. At the same time, it is less labour intensive in comparison to standard morphometry based on a large number of measurements of various body parts [6].

Honey bees inhabiting areas of present-day Romania have been used by local people since ancient times, and beekeeping has been an important occupation since the Middle Ages [49,50]. Even between the Second World War and 1989, when Romania was isolated from other countries, beekeeping continued to develop, supported by the Romanian Beekeepers Association, which also included a research and breeding infrastructure. The concept of local bee preservation and import prohibition is relatively old in Romania [51]. Based on melliferous flora and climatic conditions, local ecotypes of honey bees were designated in Romania [52,53,54,55]. A protection and breeding programme of local bees was established, and the importing of non-native bees was state controlled by ministerial permission, Marza 1965, 1968, 1970 from [52]. In the situation of limited cross-border travel and strict border control, imports were probably much lower in comparison to other countries of Western and even Eastern Europe. Transhumance and hybridisation within the country were widespread and involved migratory beekeeping and queen trading between the intra- and extra-Carpathian areas. Up to 30–40% of beekeepers in Romania practice migratory beekeeping; they can travel with their bees within the country, even up to 600 km. Intensive beekeeping is practised in the whole country, except the highest parts of Carpathians, and is based mainly on local bees [52]. Most Romanian beekeepers consider local bees as suitable for beekeeping and understand the need for their protection. Today, beekeeping legislation (Law 383/2013) designates the protection of the native subspecies *A. m. carpatica* and regulates the breeding programme for queen trading. According to this legislation, the introduction of non-native bees is under state control. Despite conservation efforts, in recent years, the illegal introduction of non-native honey bees could have occurred, as in other European countries [56].

The aim of this study was to verify whether wing venation in honey bees in Romania has changed over the last four decades. We investigated the temporal changes in two subpopulations separated by the Carpathian Mountains, which are a significant geographical barrier. We expect that the introduction of non-native subspecies affected the studied subpopulations, and that wing venation has changed over time. Moreover, we expect that the two subpopulations separated by the Carpathians will, over time, become more similar to each other. We also verify how wing venation was influenced by annual temperatures, as it has been earlier suggested that climate rather than geographic barriers are responsible for shaping geographic variation of honey bees in Romania [44].

## 2. Materials and Methods

**Figure 1:**
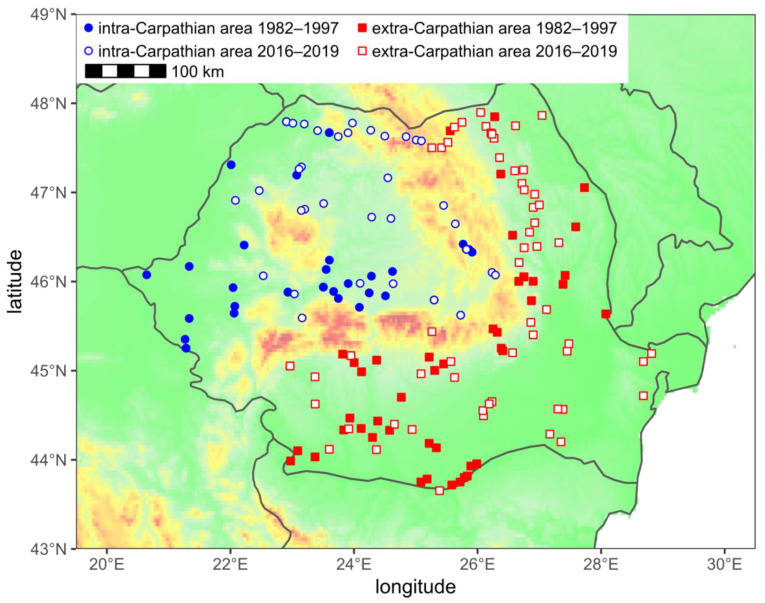
A map of the study area. The markers indicate the locations from which the samples were collected. The background colours from green to red indicate increasing elevation.

In this study, we used 6498 wings of honey bee workers, which represented 197 samples. Of these, 166 samples (5287 wings) were collected from colonies, and 31 samples (1211 wings) were collected from flowers (Supplementary Tables S1 and S2. The study area was divided along the highest ridges of the Carpathians into two areas—intra-Carpathian (*n* = 88) in the northwest and extra-Carpathian (*n* = 109) in the south and east of the country (Figure 1).

This study covers samples collected between 1982 and 2019 (Tables S1 and S2. The samples collected between the years 1982 and 1997 were obtained from the collection of specimens used for classic morphometry, preserved in the Institute for Beekeeping Research and Development, Bucharest. These samples consisted of forewings, dry-mounted between two microscopic slides. Each mounting represented one colony. The number of wings per sample from the years 1982 to 1997 was, on average, 34.2. The samples collected in 2016 (by E.C., A.S., G.O.V. and D.C.) were obtained from colonies managed by beekeepers and consisted of workers preserved in alcohol. In those samples, there were on average 28.1 wings per colony. The samples collected in 2019 (by A.T.) were obtained from flowers and consisted of workers preserved in alcohol. In those samples, there were on average 39.1 wings per sample. Samples from 2019 represented locations that were spaced no closer than 10 km from each other. The samples collected from flowers most probably consisted of workers originating from more than one colony or apiary. The samples were collected during the summer; therefore, the results can be affected to some degree by migratory beekeeping.

According to the year of collection, the samples were divided into two groups: the samples collected before 2000 (between the years 1982 and 1997) and the samples collected after 2000 (between the years 2016 and 2019). The year 2000 was chosen to obtain two groups of similar sizes. This date coincides also with political changes, which resulted in easier international travel and accession of Romania to the EU, as free movement and trading could have an impact on the honeybee population. The sample size of the groups before and after 2000 was 102 and 95, respectively. Four area/time groups were analysed: intra-Carpathian before 2000 (*n* = 52), intra-Carpathian after 2000 (*n* = 36), extra-Carpathian before 2000 (*n* = 50) and extra-Carpathian after 2000 (*n* = 59).

Both right and left forewings were measured. The difference between the left and right wings is relatively small and has little effect on subspecies identification [57]. Wing images were obtained using a USB camera equipped with a 25 mm lens (FL-CC2514-2M, Ricoh). The resolution of the image was 2400 dpi. The wings were measured according to the methodology of geometric morphometrics. On each wing image, 19 landmarks (Figure S1) were indicated using IdentiFly software (for more details, see [58]). Each landmark was described by two coordinates, which gives 38 variables. The landmark coordinates were aligned using generalised Procrustes analysis [59] in MorphoJ 1.06 software [60]. Next, the aligned coordinates were subjected to principal component analysis in order to obtain 34 PC scores, which were later referred to as “wing shape.” Wing size was measured as a natural logarithm of centroid size. Centroid size was calculated as the square root of the sum of the squared distances between the centres of the forewings and the landmarks.

Latitude, longitude and average yearly temperature were determined for each location. Temperature data were obtained from http://www.worldclim.org/, accessed on 15 December 2020, [61]. Mean monthly temperatures at a spatial resolution of 30 s were averaged out in order to calculate the yearly temperature to be used in the analysis. The landmark coordinates were averaged out within colonies or locations (in the case of samples collected from flowers). Wing shapes were compared between area/time groups using the multivariate analysis of variance (MANOVA). Relationships between wing shape and altitude, latitude and altitude and annual temperatures were analysed using multivariate regression. Relationships between two univariate variables (for example temperature and latitude) were analysed with the Pearson correlation. Wing size and cubital index were compared between area/time groups using the analysis of variance (ANOVA). Differentiation between area/time groups and two neighbouring subspecies was analysed using canonical variate analysis (CVA). From the later analysis, we also obtained Mahalanobis distances between groups. In order to compare the possible effect of temperature and the Carpathian Mountains on wing shape, the samples were grouped into intra- and extra-Carpathian or those with an annual temperature above and below 9 °C. Next, the two pairs of groups were analysed with CVA in order to determine the classification rate and Mahalanobis distance between wing shape from different areas or temperatures. The success of the differentiation was verified by leave-one-out cross-validation using Past 3.11 software [62]. All other statistical analyses were performed with Statistica v. 13 software. Wing shape data were compared with reference samples obtained from the Morphometric Bee Data Bank in Oberursel, Germany. These samples consisted of wing images from 15 colonies of *A. m. carnica* and 2 colonies of *A. m. macedonica*.

In order to identify bees from Romania, IdentiFly software should be used, as explained by Nawrocka et al. [58]. The identification data related to this study are provided in two files: “apis-mellifera-carpatica-classification.dw.xml” and “apis-mellifera-carpatica-subpopulation-classification.dw.xml”. The first file allows discrimination between *A. m. carpatica* and other subspecies from Lineage C and Lineages A, M and O. The second file allows discrimination between *A. m. carnica*, intra-Carpathian *A. m. carpatica* and extra-Carpathian *A. m. carpatica*. It is recommended to start identification with the first file and, if the sample is classified as *A. m. carpatica*, continue with the second file in order to discriminate between subpopulations. The software and the identification data can be downloaded from http://drawwing.org/identifly, accessed on 1 June 2021. Both identification files related to this study can be found in the “xml” folder of IdentiFly version 1.6 or higher.

## 3. Results

### 3.1. Wing Shape

**Figure 2:**
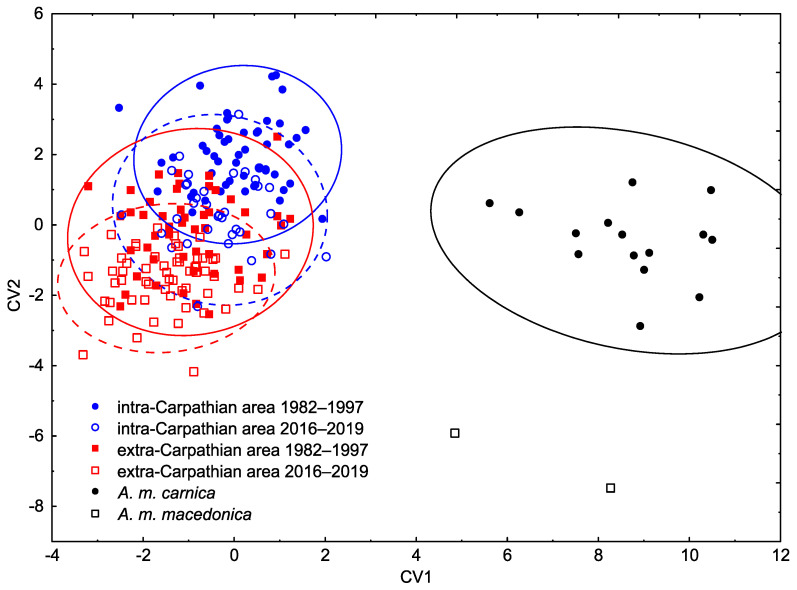
Discrimination between the areas and the reference samples, based on a canonical variate analysis of wing shape.

Canonical variate analysis revealed that the wing shapes (represented by 34 principal scores) of bees from Romania differed markedly from those of *A. m. carnica* and *A. m. macedonica* samples obtained from the Oberursel Data Bank (Figure 2, Table 1). There were also significant differences between the intra- and extra-Carpathian areas (two-factor MANOVA, area factor: Wilks’ lambda = 0.35, *p* < 0.0001) and between bees sampled before and after the year 2000 (two-factor MANOVA, time factor: Wilks’ lambda = 0.39, *p* < 0.0001). Interaction between the two factors was also significant (although less markedly) (two-factor MANOVA, area * time interaction: Wilks’ lambda = 0.75, *p* = 0.0340).

**Figure 3:**
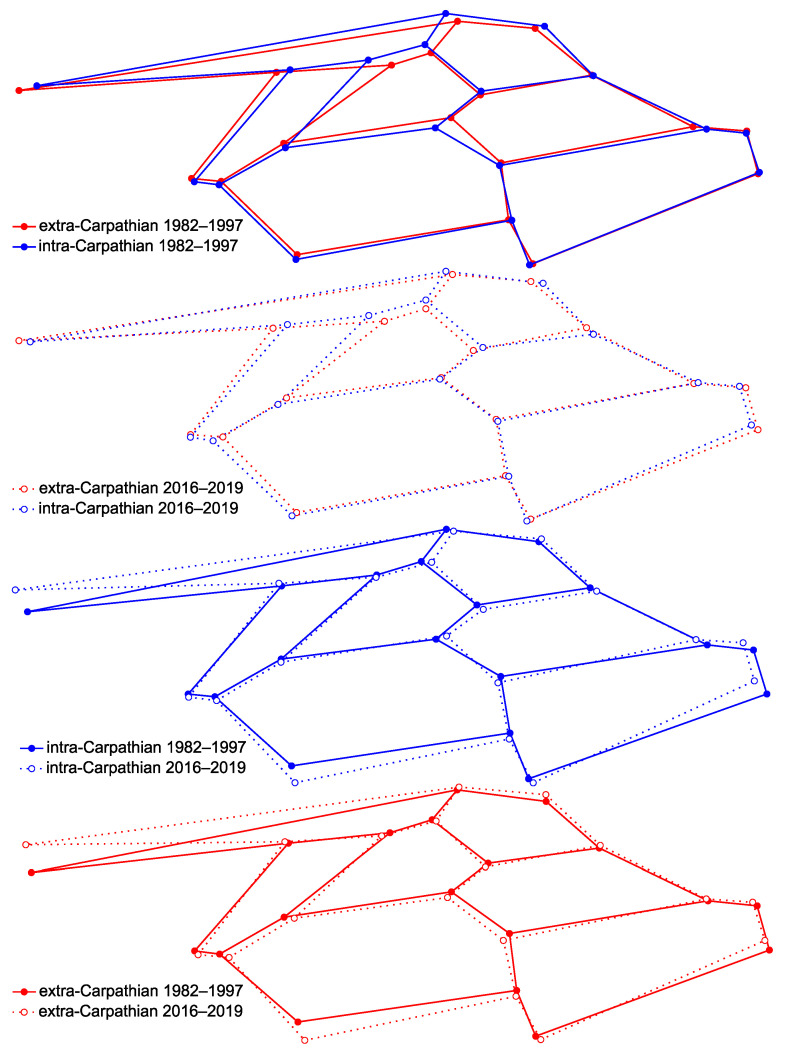
Differences between the intra- and extra-Carpathian groups of samples collected in the years 1982–1997 and 2016–2019. The differences were increased by 5-fold.

In general, the honey bees from Romania were more similar to *A. m. carnica* than to *A. m. macedonica* (Table 1). Moreover, the similarity to *A. m. carnica* was higher in the intra-Carpathian area than in the extra-Carpathian area (Table 1). On the other hand, the similarity to *A. m. macedonica* was higher in the extra-Carpathian area than in the intra-Carpathian area (Table 1). The differences between the intra- and extra-Carpathian areas were mainly located in the mid and anterior parts of the wing (Figure 3 and Figure S2). On the other hand, the differences between the historical and recent samples were mainly in the distal, posterior and proximal parts of the wing (Figure 3).

The differences between the intra- and extra-Carpathian samples collected before 2000 (9.28, Table 1) were greater in comparison to the recent samples (8.00, Table 1). However, both these differences were greater than the differences between the historical and recent samples within the intra- and extra-Carpathian areas (6.92 and 7.79, respectively, Table 1). This shows that, over time, the intra-Carpathian population became less similar to the *A. m. carnica* reference and more similar to the *A. m. macedonica* reference (Table 1). On the other hand, the extra-Carpathian population over time became less similar to both the *A. m. carnica* and *A. m. macedonica* references (Table 1).

When historical and contemporary samples were combined within areas, the squared Mahalanobis distance between the areas was 9.02. Linear discriminant analysis allowed us to correctly classify, with cross-validation, 85.28% of the colonies from the intra- and extra-Carpathian areas. The misclassified colonies were located not only near boundaries between the two areas but also within them (Figure S3).

When the samples were grouped according to the annual temperature (below and above 9 °C), the squared Mahalanobis distance between the samples with low and high temperatures was 1.79. Linear discriminant analysis correctly classified, with cross-validation, 58.88%, of the colonies with temperatures below or above 9 °C. The annual temperature significantly negatively correlated with the latitude (Pearson correlation: r = −0.54, *p* < 0.0001), longitude (Pearson correlation: r = −0.17, *p* = 0.0201) and altitude (Pearson correlation: r = −0.93, *p* < 0.0001).

There was a significant relationship between wing shape and latitude (multivariate regression: Wilks’ lambda = 0.39, *p* < 0.0001), longitude (multivariate regression: Wilks’ lambda = 0.60, *p* < 0.0001), altitude (multivariate regression: Wilks’ lambda = 0.69, *p* = 0.0011) and average annual temperature (multivariate regression: Wilks’ lambda = 0.62, *p* < 0.0001).

**Figure 4:**
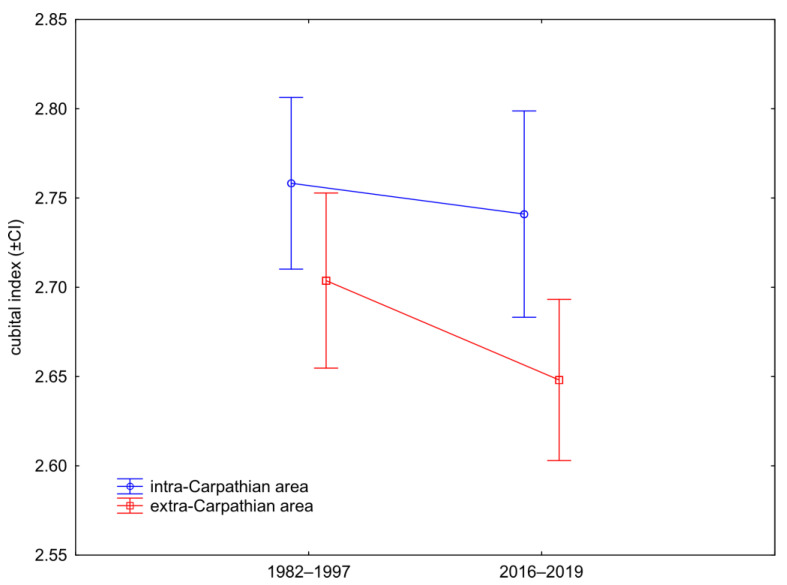
Differences in the cubital index (±95% confidence intervals) between the intra- and extra-Carpathian samples collected in the years 1982–1997 and 2016–2019.

The cubital index differed markedly between the intra- and extra-Carpathian areas (two-factor ANOVA, area factor: F(1,193) = 8.37, *p* = 0.0042), but there was no significant differences between the bees collected before and after the year 2000 (two-factor ANOVA, time factor: F(1,193) = 2.04, *p* = 0.1539). The interaction between the two factors was also not significant (two-factor ANOVA, area * time interaction: F(1,193) = 0.56, *p* = 0.4516, Figure 4).

### 3.2. Wing Size

**Figure 5:**
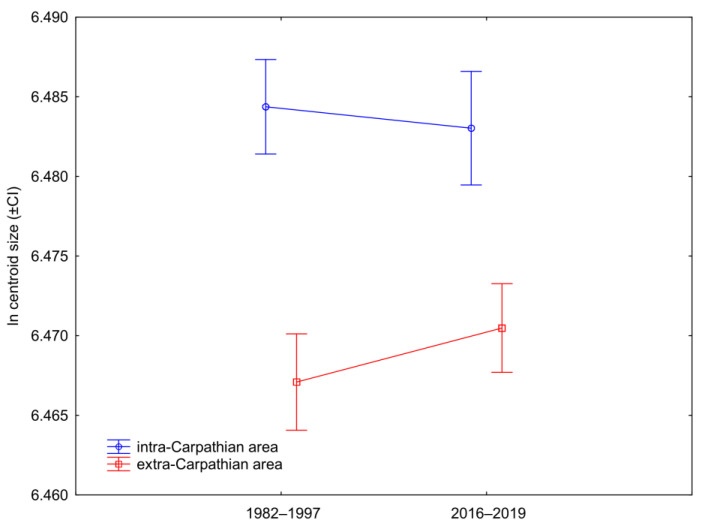
Differences in wing size (±95% confidence intervals) between the intra- and extra-Carpathian samples collected in the years 1982–1997 and 2016–2019.

Wing size (represented by centroid size) differed significantly between the intra- and extra-Carpathian areas (two-factor ANOVA: F(1,193) = 90.13; *p* < 0.0001; Figure 5) but not between the historical and recent samples (two-factor ANOVA: F(1,193) = 0.42; *p* = 0.5153; Figure 5). The interaction between the two factors was also not significant (two-factor ANOVA, area * time interaction: F(1,193) = 2.27; *p* = 0.1329; Figure 5).

**Figure 6:**
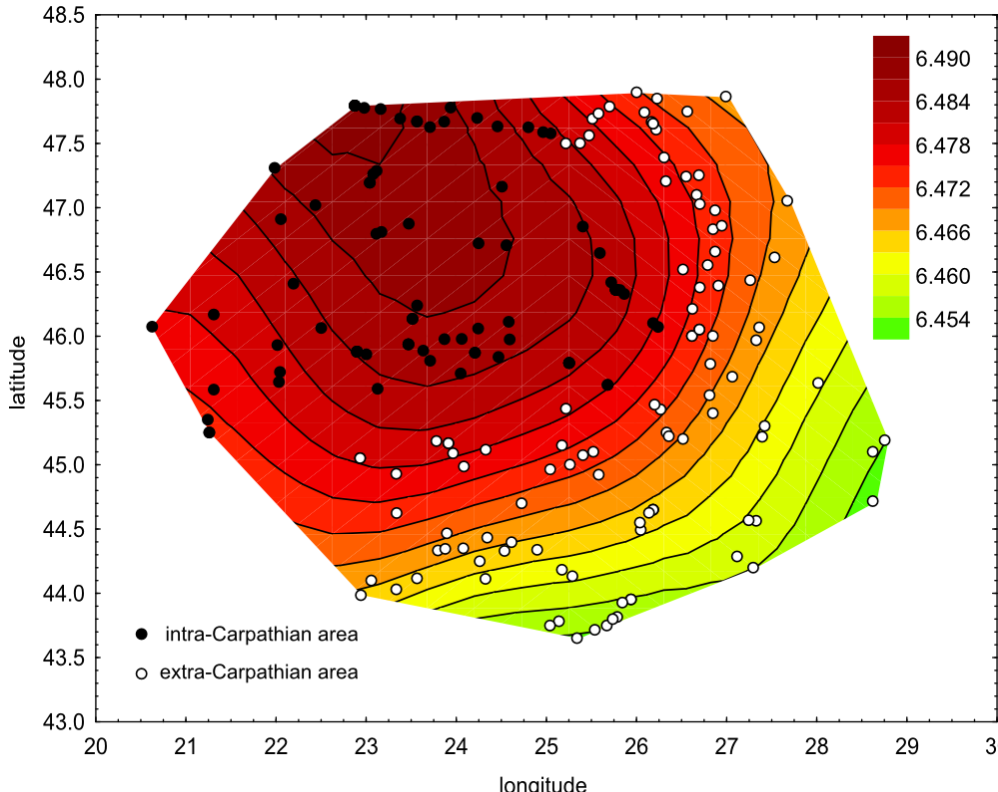
Wing size interpolated over the longitude and latitude of the study area. The colours from green to red represent a natural logarithm of centroid size.

The largest wing size was in the northwest part of the study area, and it decreased towards the southeast (Figure 6). The wing size was significantly positively correlated with the latitude (Pearson correlation: r = 0.45, *p* < 0.0001) and altitude (Pearson correlation: r = 0.33, *p* < 0.0001); on the other hand, it significantly negatively correlated with longitude (Pearson correlation: r = −0.37, *p* < 0.0001) and average annual temperature (Pearson correlation: r = −0.34, *p* < 0.0001).

## 4. Discussion

The data presented here clearly show that, in Romania, the shape of forewing venation has changed markedly during the last four decades. The most likely explanation of those changes is the introduction by beekeepers of non-native bees, which hybridised with native ones. Some homogenisation of wing shape within the study area was also observed, which could be explained by migratory beekeeping and queen trading activities. The Mahalanobis distance between wing shape in the two investigated areas was higher before than after 2000. We suspect that similar or higher temporal changes also occurred in other parts of Europe, as there are numerous studies indicating signs of introgression in European honey bee populations [21,23]. Most of these studies are based on the assumption that, originally, the native population was characterised by a particular mitotype or microsatellite pattern. Rarely, there were direct comparisons between the historical and contemporary samples from mainland Europe [36,37]. Such studies are essential for monitoring not only the presence but also the dynamics of the hybridisation process.

Despite the marked temporal changes in the investigated Romanian honeybee subpopulation there was still a clear difference between the honeybees originated from intra- and extra-Carpathian areas. As expected, the similarity to *A. m. carnica* was higher in the intra-Carpathian area, and to *A. m. macedonica* was higher in the extra-Carpathian area (Table 1). This is, to some degree, in agreement with conclusions of earlier studies that *A. m. carnica* and *A. m. macedonica* would be in intra- and extra-Carpathian areas, respectively [6]. The observed differences indicate that, in both of these areas, a large part of the original genetic patrimony was still preserved. This is the first geometric morphometric comparison of honey bee forewings in Romania; therefore, we were not able to directly compare most of our results with earlier studies. In order to do this, we calculated the cubital index. As expected, it was higher in the intra-Carpathian area than in the extra-Carpathian area (Figure 4). This confirms earlier reports by Fisteag [39] and Foti [40] but differs from Coroian et al. ([44], Table S6), where the differences in cubital index between the intra- and extra-Carpathian areas were not significant. The cubital index in honeybees collected from intra-Carpathian Romania (2.75) was markedly higher than in Croatia and Slovenia (2.46) [63] or former Yugoslavia (2.51) ([6], Table 14.2). In this context, it is surprising that Ruttner [6] considered bees from former Yugoslavia and intra-Carpathian Romania as one subspecies—*A. m. carnica*. On the other hand, Ruttner [6] considered bees from intra- and extra-Carpathian Romania (cubital index: 2.75, 2.67, respectively) as two subspecies: *A. m. carnica* and *A. m. macedonica*, respectively. It should be stressed, however, that the designation of the distribution area of the two subspecies was based on 13 out of 36 variables used in standard morphometry [6], and among the selected variables cubital index was not present. In general, the results presented here suggest that Ruttner’s concept, regarding the subspecies distribution in Romania, needs to be reviewed; however, this requires more data from a larger area. Specifically, the concept of *A. m. carpatica* [40] should be reconsidered. The relatively large difference between the Romanian bees and both the *A. m. carnica* and *A. m. macedonica* bees references suggests that they are distinct. In this study, we used only two colonies of *A. m. macedonica*; therefore, the results related to this subject should be considered preliminary. The distinctness of *A. m. carpatica* when compared with *A. m. carnica* and *A. m. macedonica* was also confirmed by a recent study based on single-nucleotide polymorphism ([45], Figure S1). However, in another study based on microsatellites, bees from Romania were similar to those from Slovenia ([44], Figure S3).

Wing size differed markedly within the study area. In the intra-Carpathian area, it was larger in comparison to the extra-Carpathian area. The spatial profile of wing size (Figure 6) corresponded to the shape of the Carpathian Mountains (Figure 1). The observed differences agree with earlier studies. It was reported that the bees in Transylvania were bigger than in the Danube valley [39,40,52]. As expected, the wing sizes in the intra-Carpathian samples (ln centroid size at 2400 dpi: 6.48) were similar to those in Croatia and Slovenia (ln centroid size at 2400 dpi: 6.49) [63].

In an earlier study, it was suggested that the geographical variation of honey bees in Romania was better explained by the average temperature (below or above 9 °C) than isolation by the Carpathians [44]. According to this suggestion, the two hypothetical warm and cold climate ecotypes would not be separated by any geographical barriers, and it is difficult to explain how they could be maintained. The results obtained in this study were contrary to the suggested climate ecotypes. The grouping of the samples into intra- and extra-Carpathian areas resulted in a higher Mahalanobis distance between the groups and a higher identification rate in comparison to the grouping based on temperatures above or below 9 °C. Moreover, both wing shape and size depended much more on latitude than on average temperature. However, it is difficult to separate the effect of the two variables because they are highly correlated with each other. In this situation, it cannot be completely ruled out that the observed temporal changes in wing venation were in part related to climate change.

The data presented here show that, in Romania, relatively well-preserved native honey bees, that deserve to be protected, were still present. The need for conservation measures is urgent because relatively rapid changes were observed, which are most probably related to hybridisation. The observed differences between the intra- and extra-Carpathian subpopulations can be explained by adaptation to the local environment. These adaptations are beneficial for beekeeping, as they increase the resilience and survival of honey bee colonies [24]. The protection of local biodiversity should involve controlling the introduction of non-native bees and the elimination of non-native bees from the populations by selective breeding. Both these procedures require differentiation between native and non-native bees. Here we provide an identification method for both the local Romanian subpopulations. The provided method can be used only to discriminate between the intra- and extra-Carpathian bees and between Romanian and non-Romanian bees; it does not allow precisely indicating from which geographic region the non-Romanian bees were introduced. For this, a much larger data set would be required, covering a large number of geographic regions.

## 5. Conclusions

Our results suggest that, in Romania, there is a risk of hybridisation between native and non-native bees. Despite the observed temporal changes, the local honey bee, *A. m. carpatica,* is relatively well preserved. Two distinct subpopulations separated by the Carpathians are still present. These subpopulations deserve protection. We provide identification data that can be used in the monitoring, breeding and conservation of *A. m. carpatica*.

## Figures and Tables

**Table 1:** Squared Mahalanobis distances (lower triangle) between the investigated groups and the significance of pairwise comparisons (upper triangle). ***—*p* < 0.0001.

Groups	Intra-Carpathian 1982–1997	Extra-Carpathian 1982–1997	Intra-Carpathian 2016–2019	Extra-Carpathian 2016–2019	*A. m. carnica*	*A. m. macedonica*
intra-Carpathian 1982–1997		***	***	***	***	***
extra-Carpathian 1982–1997	9.28		***	***	***	***
intra-Carpathian 2016–2019	6.92	11.59		***	***	***
extra-Carpathian 2016–2019	15.68	7.79	8.00		***	***
*A. m. carnica*	81.03	96.22	85.33	104.45		***
*A. m. macedonica*	151.34	135.31	140.40	136.41	92.67	

## Data Availability

The data presented in this study are available on request from the corresponding author.

## Supplementary Materials

The following are available online at https://www.mdpi.com/article/10.3390/insects12060542/s1, Figure S1: Schematic of the fore wing of a honey bee worker with landmarks marked with black dots and numbers. Figure S2: Differences between the forewings of honey bees collected in intra- and extra-Carpathian Romania and *A. m. carnica* and *A. m. macedonica*. The differences were increased by 5-fold. Figure S3: Location of samples correctly and incorrectly classified as belonging to the intra- and extra-Carpathian areas. Table S1: Information about honey bee samples used for the analysis of the temporal variation of honey bees in the intra- and extra-Carpathian areas of Romania. Table S2: Number of samples and wings used for the analysis of temporal variation of honey bees in the intra- and extra-Carpathian areas of Romania.
