# Supplementary material for: Historical Changes in Honey Bee Wing Venation in Romania
Source: Insects. 2021 Jun 10;12(6):542. doi: 10.3390/insects12060542 (PMC8230453; doi:10.3390/insects12060542)
Supplement: Supplementary file 1 [file insects-12-00542-s001.zip › Table-S1.pdf]

Supplementary Table 1. Information about honey bee samples used for the analysis of temporal variation of honey bees in intra- and extra-Carpathian area of Romania.

no	latitude	longitude	altitude	region	year	source
1	46.2371	23.585	361	intra-Carpathian	1982	colony
2	45.8861	23.6530	360	intra-Carpathian	1982	colony
3	45.9351	23.4875	283	intra-Carpathian	1982	colony
4	45.9351	23.4875	283	intra-Carpathian	1982	colony
5	45.9351	23.4875	283	intra-Carpathian	1982	colony
6	46.1333	23.5333	294	intra-Carpathian	1982	colony
7	46.1333	23.5333	294	intra-Carpathian	1982	colony
8	44.7	24.75	252	extra-Carpathian	1995	colony
9	44.9634	25.0692	457	extra-Carpathian	2016	colony
10	45.4347	25.2448	1276	extra-Carpathian	2019	flowers
11	46.4062	22.2058	178	intra-Carpathian	1987	colony
12	46.1666	21.3166	107	intra-Carpathian	1996	colony
13	46.4333	27.3	312	extra-Carpathian	2016	colony
14	46.391	26.9444	199	extra-Carpathian	2016	colony
15	46.5166	26.55	387	extra-Carpathian	1990	colony
16	46.6556	26.9083	194	extra-Carpathian	2019	flowers
17	46.3771	26.7376	266	extra-Carpathian	2019	flowers
18	46.2106	26.6549	267	extra-Carpathian	2019	flowers
19	46.5510	26.8254	312	extra-Carpathian	2019	flowers
20	46.9076	22.065	252	intra-Carpathian	2016	colony
21	47.0167	22.45	249	intra-Carpathian	2016	colony
22	47.3063	21.9953	102	intra-Carpathian	1982	colony
23	47.1601	24.5306	383	intra-Carpathian	2016	colony
24	45.2166	27.4333	35	extra-Carpathian	2016	colony
25	45.3005	27.4613	20	extra-Carpathian	2016	colony
26	47.7443	26.5983	180	extra-Carpathian	2016	colony
27	47.8604	27.0275	183	extra-Carpathian	2016	colony
28	45.79	25.28	619	intra-Carpathian	1996	colony
29	45.79	25.28	619	intra-Carpathian	1997	colony
30	45.79	25.28	619	intra-Carpathian	1997	colony
31	45.62	25.7097	707	intra-Carpathian	2016	colony
32	45.62	25.7097	707	intra-Carpathian	2016	colony
33	45.79	25.28	619	intra-Carpathian	2016	colony
34	45.25	26.3666	294	extra-Carpathian	1996	colony
35	45.221	26.3919	254	extra-Carpathian	1996	colony
36	45.429	26.3006	506	extra-Carpathian	1996	colony
37	45.4666	26.2333	842	extra-Carpathian	1996	colony
38	45.2	26.55	235	extra-Carpathian	2016	colony
39	45.4	26.8833	334	extra-Carpathian	2016	colony

40	44.4924	26.0791	88	extra-Carpathian	2016	colony
41	46.8082	23.1872	662	intra-Carpathian	2016	colony
42	46.794	23.13	672	intra-Carpathian	2016	colony
43	46.8726	23.4917	538	intra-Carpathian	2016	colony
44	44.2	27.3333	11	extra-Carpathian	2016	colony
45	44.2868	27.1533	28	extra-Carpathian	2016	colony
46	46.1	26.2166	676	intra-Carpathian	2016	colony
47	46.070	26.2725	588	intra-Carpathian	2016	colony
48	45.000	25.2889	371	extra-Carpathian	1996	colony
49	45.15	25.2	625	extra-Carpathian	1996	colony
50	45.0737	25.4342	398	extra-Carpathian	1996	colony
51	45.1	25.55	436	extra-Carpathian	2016	colony
52	44.9221	25.61	268	extra-Carpathian	2016	colony
53	44.0308	23.3525	57	extra-Carpathian	1990	colony
54	43.9858	22.9575	54	extra-Carpathian	1990	colony
55	44.3333	23.8166	110	extra-Carpathian	1990	colony
56	44.1	23.0733	78	extra-Carpathian	1990	colony
57	44.3457	23.8985	185	extra-Carpathian	2016	colony
58	44.1166	23.5833	87	extra-Carpathian	2016	colony
59	45.0505	22.9488	281	extra-Carpathian	1995	colony
60	45.1833	23.8	669	extra-Carpathian	1997	colony
61	45.0505	22.9488	281	extra-Carpathian	2016	colony
62	44.929	23.3522	293	extra-Carpathian	2016	colony
63	46.0666	27.4	97	extra-Carpathian	1990	colony
64	45.6346	28.0578	17	extra-Carpathian	1990	colony
65	45.9666	27.3666	183	extra-Carpathian	1990	colony
66	43.8166	25.8166	21	extra-Carpathian	1994	colony
67	43.75	25.7	37	extra-Carpathian	1994	colony
68	43.8	25.7666	23	extra-Carpathian	1994	colony
69	43.9529	25.97	22	extra-Carpathian	1994	colony
70	43.9529	25.97	22	extra-Carpathian	1994	colony
71	43.9284	25.8736	85	extra-Carpathian	1994	colony
72	45.8780	22.9144	190	intra-Carpathian	1997	colony
73	45.8780	22.9144	190	intra-Carpathian	1997	colony
74	45.8780	22.9144	190	intra-Carpathian	1997	colony
75	45.8780	22.9144	190	intra-Carpathian	1997	colony
76	45.8780	22.9144	190	intra-Carpathian	1997	colony
77	45.5898	23.1442	679	intra-Carpathian	2016	colony
78	46.062	22.5152	450	intra-Carpathian	2016	colony
79	45.8573	23.0186	192	intra-Carpathian	2019	flowers
80	46.36	25.8	681	intra-Carpathian	1986	colony
81	46.36	25.8	681	intra-Carpathian	1987	colony
82	46.36	25.8	681	intra-Carpathian	1987	colony
83	46.36	25.8	681	intra-Carpathian	1987	colony
84	46.3265	25.895	747	intra-Carpathian	1996	colony

85	46.358	25.804	681	intra-Carpathian	1996	colony
86	46.4166	25.75	682	intra-Carpathian	1996	colony
87	46.3568	25.8469	773	intra-Carpathian	1996	colony
88	46.358	25.804	681	intra-Carpathian	1996	colony
89	46.644	25.6228	765	intra-Carpathian	2016	colony
90	46.358	25.804	681	intra-Carpathian	2016	colony
91	46.85	25.4333	732	intra-Carpathian	2016	colony
92	44.65	26.2166	86	extra-Carpathian	1994	colony
93	44.65	26.2166	86	extra-Carpathian	1994	colony
94	44.65	26.2166	86	extra-Carpathian	2016	colony
95	44.55	26.07	95	extra-Carpathian	2016	colony
96	44.6257	26.1711	89	extra-Carpathian	2016	colony
97	44.5638	27.3661	23	extra-Carpathian	2016	colony
98	44.5682	27.2831	25	extra-Carpathian	2016	colony
99	47.0504	27.7133	240	extra-Carpathian	1988	colony
100	47.2387	26.5849	269	extra-Carpathian	2019	flowers
101	47.0966	26.7020	253	extra-Carpathian	2019	flowers
102	47.2494	26.7272	231	extra-Carpathian	2016	colony
103	44.624	23.3561	213	extra-Carpathian	2016	colony
104	47.6666	23.5833	471	intra-Carpathian	1986	colony
105	47.6666	23.5833	471	intra-Carpathian	1986	colony
106	47.6898	23.3949	152	intra-Carpathian	2019	flowers
107	47.6227	23.7280	372	intra-Carpathian	2019	flowers
108	47.6655	23.8866	956	intra-Carpathian	2019	flowers
109	47.7744	23.9581	419	intra-Carpathian	2019	flowers
110	47.6936	24.2546	393	intra-Carpathian	2019	flowers
111	47.6282	24.4815	613	intra-Carpathian	2019	flowers
112	47.6213	24.8255	1055	intra-Carpathian	2019	flowers
113	46.72	24.27	379	intra-Carpathian	2016	colony
114	46.7046	24.5814	385	intra-Carpathian	2016	colony
115	47.2025	26.3586	379	extra-Carpathian	1989	colony
116	46.9763	26.9078	222	extra-Carpathian	2019	flowers
117	46.8293	26.8848	174	extra-Carpathian	2019	flowers
118	46.8564	26.9829	259	extra-Carpathian	2016	colony
119	47.0249	26.7378	306	extra-Carpathian	2016	colony
120	44.3296	24.5592	159	extra-Carpathian	1991	colony
121	44.4333	24.3667	148	extra-Carpathian	1991	colony
122	44.4666	23.9166	194	extra-Carpathian	1990	colony
123	44.35	24.0994	127	extra-Carpathian	1994	colony
124	44.2495	24.283	125	extra-Carpathian	1994	colony
125	44.3976	24.6375	173	extra-Carpathian	2016	colony
126	44.1125	24.3472	107	extra-Carpathian	2016	colony
127	46.1101	24.6067	533	intra-Carpathian	1987	colony
128	45.8368	24.4922	461	intra-Carpathian	1987	colony
129	45.87	24.23	544	intra-Carpathian	1997	colony

130	46.0579	24.2658	450	intra-Carpathian	1987	colony
131	45.8073	23.73	822	intra-Carpathian	1997	colony
132	45.9751	23.8892	425	intra-Carpathian	1987	colony
133	45.7085	24.0706	543	intra-Carpathian	1997	colony
134	45.87	24.23	544	intra-Carpathian	1997	colony
135	45.9730	24.6172	481	intra-Carpathian	2016	colony
136	45.9771	24.0825	464	intra-Carpathian	2016	colony
137	47.1911	23.0572	291	intra-Carpathian	1987	colony
138	47.1911	23.0572	291	intra-Carpathian	1982	colony
139	47.2833	23.1333	286	intra-Carpathian	2016	colony
140	47.2582	23.0942	257	intra-Carpathian	2016	colony
141	47.79	22.89	122	intra-Carpathian	1986	colony
142	47.79	22.89	122	intra-Carpathian	1986	colony
143	47.79	22.89	122	intra-Carpathian	1986	colony
144	47.79	22.89	122	intra-Carpathian	1986	colony
145	47.7901	22.8911	124	intra-Carpathian	2019	flowers
146	47.79	22.89	122	intra-Carpathian	1986	colony
147	47.7634	23.1780	134	intra-Carpathian	2019	flowers
148	47.79	22.89	122	intra-Carpathian	2016	colony
149	47.7724	22.9947	125	intra-Carpathian	2016	colony
150	47.6876	25.54	775	extra-Carpathian	1988	colony
151	47.8449	26.2608	276	extra-Carpathian	1988	colony
152	47.5839	24.9900	1034	intra-Carpathian	2019	flowers
153	47.5742	25.0741	955	intra-Carpathian	2019	flowers
154	47.4966	25.2434	1062	extra-Carpathian	2019	flowers
155	47.4976	25.4014	815	extra-Carpathian	2019	flowers
156	47.5582	25.5028	803	extra-Carpathian	2019	flowers
157	47.7286	25.6076	871	extra-Carpathian	2019	flowers
158	47.7825	25.7308	530	extra-Carpathian	2019	flowers
159	47.8932	26.0328	405	extra-Carpathian	2019	flowers
160	47.7375	26.1200	358	extra-Carpathian	2019	flowers
161	47.6633	26.1998	393	extra-Carpathian	2019	flowers
162	47.6039	26.2507	374	extra-Carpathian	2019	flowers
163	47.3868	26.3389	333	extra-Carpathian	2019	flowers
164	47.65	26.2166	348	extra-Carpathian	2016	colony
165	47.65	26.2166	348	extra-Carpathian	2016	colony
166	45.1	28.6666	68	extra-Carpathian	2016	colony
167	45.19	28.8	2	extra-Carpathian	2016	colony
168	44.7166	28.6666	12	extra-Carpathian	2016	colony
169	45.9293	22.0264	224	intra-Carpathian	1993	colony
170	45.6425	22.0435	208	intra-Carpathian	1993	colony
171	46.0722	20.6294	82	intra-Carpathian	1995	colony
172	45.72	22.055	188	intra-Carpathian	1993	colony
173	45.25	21.2666	84	intra-Carpathian	1994	colony
174	45.25	21.2666	84	intra-Carpathian	1994	colony

175	45.5833	21.3166	89	intra-Carpathian	1994	colony
176	45.35	21.25	91	intra-Carpathian	1994	colony
177	43.78333	25.16667	67	extra-Carpathian	1994	colony
178	44.13333	25.31667	72	extra-Carpathian	1994	colony
179	43.71667	25.56667	38	extra-Carpathian	1994	colony
180	43.75	25.06667	77	extra-Carpathian	1994	colony
181	44.18333	25.2	95	extra-Carpathian	1994	colony
182	44.3385	24.9239	146	extra-Carpathian	2016	colony
183	43.65222	25.36806	23	extra-Carpathian	2016	colony
184	44.9858	24.1062	298	extra-Carpathian	1997	colony
185	45.1162	24.3497	275	extra-Carpathian	1997	colony
186	45.08853	23.9853	589	extra-Carpathian	1997	colony
187	45.16667	23.93333	694	extra-Carpathian	2016	colony
188	45.16667	23.93333	694	extra-Carpathian	2016	colony
189	46.05	26.73333	483	extra-Carpathian	1990	colony
190	46	26.88333	439	extra-Carpathian	1990	colony
191	46	26.65	584	extra-Carpathian	1997	colony
192	46	26.65	584	extra-Carpathian	1997	colony
193	45.7829	26.855	472	extra-Carpathian	1997	colony
194	46.05	26.73333	483	extra-Carpathian	1997	colony
195	45.5398	26.8464	389	extra-Carpathian	2016	colony
196	45.68333	27.1	101	extra-Carpathian	2016	colony
197	46.6107	27.5717	233	extra-Carpathian	1990	colony
