# Supplementary material for: Historical Changes in Honey Bee Wing Venation in Romania
Source: Insects. 2021 Jun 10;12(6):542. doi: 10.3390/insects12060542 (PMC8230453; doi:10.3390/insects12060542)
Supplement: Supplementary file 1 [file insects-12-00542-s001.zip › Table-S2.pdf]

Supplementary Table 2. Number of samples and wings used for the analysis of temporal variation of honey bees in intra- and extra-Carpathian area of Romania.

year	samples		wings	
	intra-Carpathian	extra-Carpathian	intra-Carpathian	extra-Carpathian
1982	9	0	320	0
1986	8	0	291	0
1987	9	0	305	0
1988	0	3	0	126
1989	0	1	0	36
1990	0	12	0	441
1991	0	2	0	55
1993	3	0	97	0
1994	4	15	137	484
1995	1	2	45	65
1996	7	7	235	200
1997	11	8	383	269
2016	24	40	660	1138
2019	12	19	514	697
