# Supplementary figures and images for: Historical Changes in Honey Bee Wing Venation in Romania
Source: Insects. 2021 Jun 10;12(6):542. doi: 10.3390/insects12060542 (PMC8230453; doi:10.3390/insects12060542)

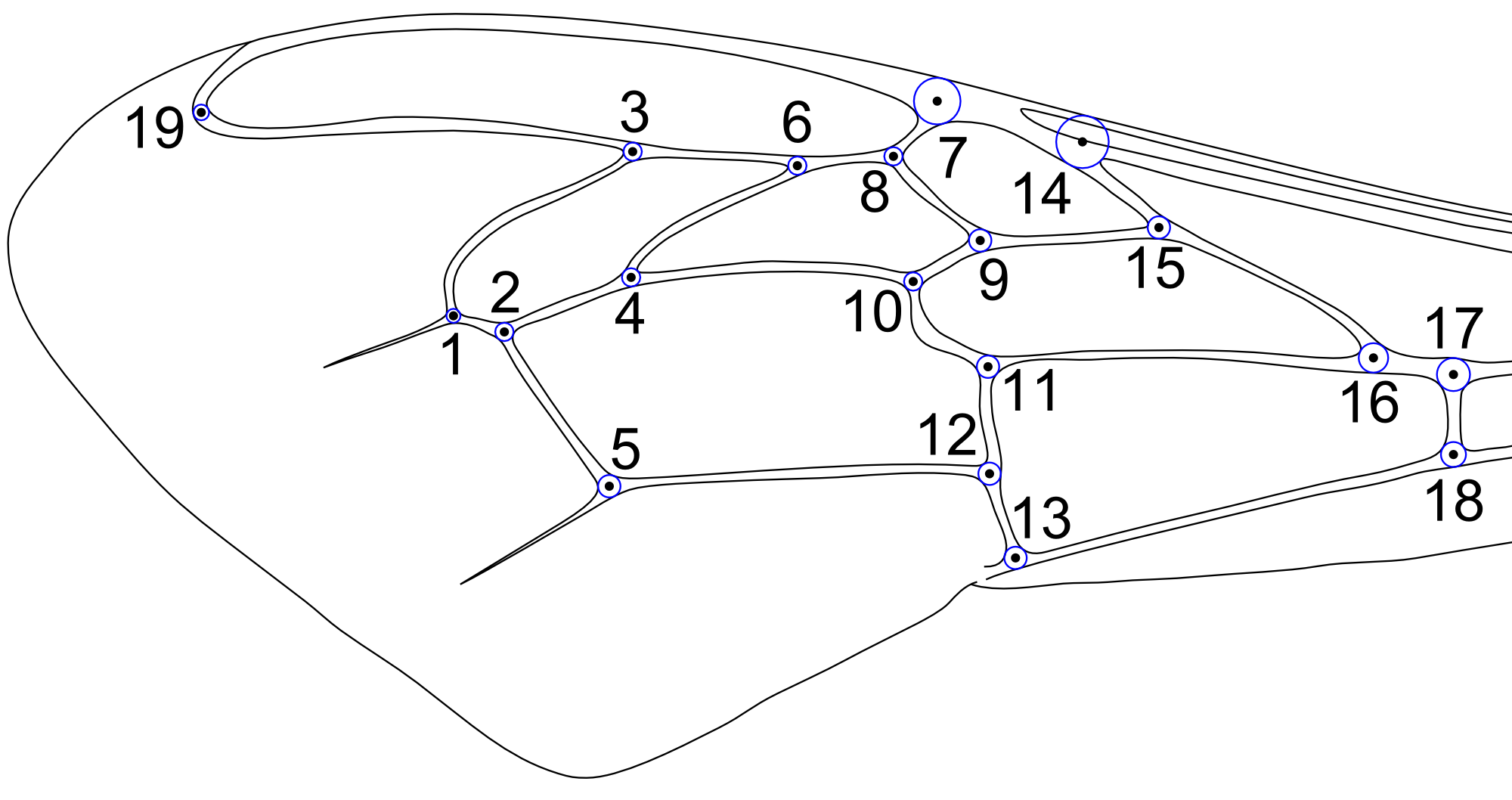

Supplement: Supplementary file 1 [file insects-12-00542-s001.zip › FigS1-apis-wing-landmarks.png]

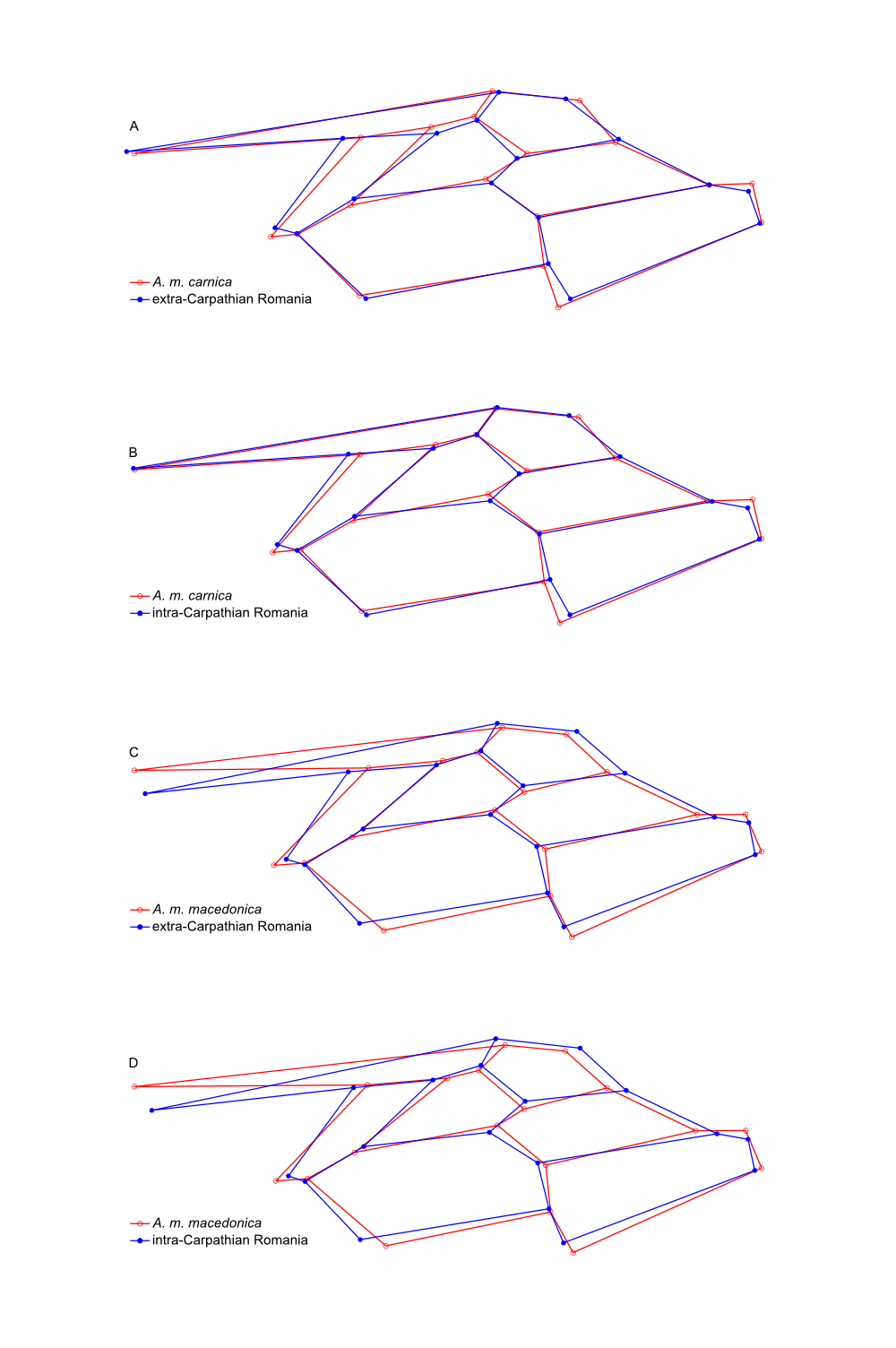

Supplement: Supplementary file 1 [file insects-12-00542-s001.zip › FigS2-supplementary-wireframe.png]

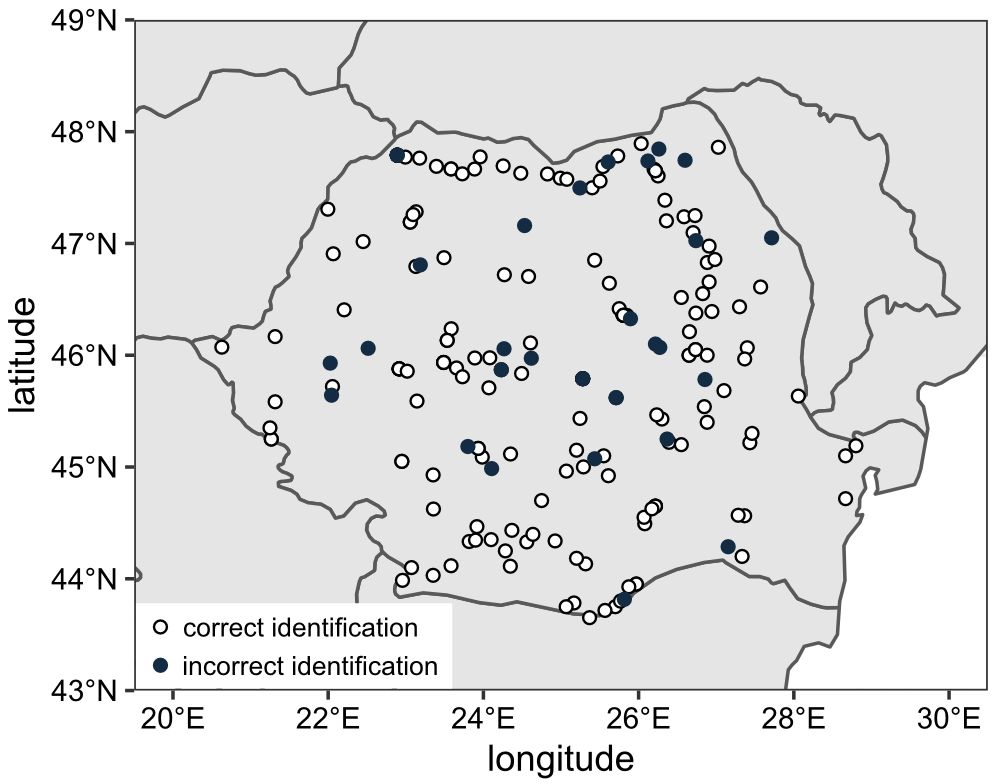

Supplement: Supplementary file 1 [file insects-12-00542-s001.zip › FigS3-in-correct-map.png]
